# Pupil-linked arousal with very light exercise: pattern of pupil dilation during graded exercise

**DOI:** 10.1186/s12576-022-00849-x

**Published:** 2022-09-24

**Authors:** Ryuta Kuwamizu, Yudai Yamazaki, Naoki Aoike, Genta Ochi, Kazuya Suwabe, Hideaki Soya

**Affiliations:** 1 Laboratory of Exercise Biochemistry and Neuroendocrinology, Faculty of Health and Sport Sciences, University of Tsukuba, Tsukuba, Ibaraki 305-8574 Japan; 2 Sports Neuroscience Division, Advanced Research Initiative for Human High Performance (ARIHHP), Faculty of Health and Sport Sciences, University of Tsukuba, Ibaraki 305-8574 Tsukuba, Japan; 3 Faculty of Health Sciences, Department of Health and Sports, Niigata University of Health and Welfare, Niigata, Niigata 950-3198 Japan; 4 Faculty of Health and Sport Sciences, Ryutsu Keizai University, Ryugasaki, Ibaraki 301-8555 Japan

**Keywords:** Incremental exercise, Arousal states, Catecholaminergic system, Pupil dilation threshold, Anaerobic threshold

## Abstract

Although it has been hypothesized that moderate to vigorous exercise immediately modulates cognition via ascending arousal system activation, such activation during very-light to light exercise has remained uncertain. Here, we aimed to uncover the exact exercise intensity necessary for ascending arousal system activation using pupillometry. The pupil diameter, psychological arousal, and ventilation during graded exercise of 26 young males were analyzed based on %${\dot{V}}_{{\text{O}}_{\text{2peak}}}$. Pupils dilated with very-light exercise compared to rest, stabilized, and then drastically increased again with moderate exercise and above. Pupil dilation with very-light exercise was positively correlated with increases in psychological arousal. Thus, we have shown that there are two phases of pupil dilation during graded exercise: one with very-light exercise coinciding with psychological arousal response, and the other with moderate exercise or above similar to the ventilation increase pattern. This unique pupil dilation pattern provides physiological evidence of ascending arousal system activation with very-light exercise.

**Supplementary Information:**

The online version contains supplementary material available at 10.1186/s12576-022-00849-x.

## Background

Physical activity has been shown to induce a change of mood, cognition, and brain health [1–4]. In particular, it has been supposed that moderate-intensity exercise and above, based on markers, such as circulating catecholamine threshold (CT), lactate threshold (LT), and ventilatory threshold (VT), which are collectively called the “anaerobic threshold”, affect cognitive performance coinciding with stress-related hormonal response [4, 5]. The neural mechanism is still unclear, but one hypothesis is the ascending arousal system, including the brain catecholamine system, arising from the locus coeruleus (LC) [6–8]. At moderate-intensity exercise and above, there is an exponential increase in lactate, hypothalamic–pituitary–adrenal axis-related stress response, and circulating catecholamine increase in an intensity-dependent manner [9, 10]. Because blood catecholamines possibly activate the LC indirectly [11, 12], it has been hypothesized that there is an exponential brain arousal response as well as an exponential peripheral catecholamines response that occur in an exercise-intensity-dependent manner [7]. This is called the catecholamine hypothesis [7, 8].

Recently, very-light-/light-intensity exercises, such as yoga, that do not achieve moderate to vigorous intensities, have become popular for improving mental health [13, 14]. According to the catecholamine hypothesis explained above, the effect of very-light-/light-intensity exercise on the ascending arousal system remains doubtful. In graded exercise, increased circulating catecholamines have not been observed during very-light-/light-intensity exercise, during which lactate also does not increase [9, 15, 16]. If blood catecholamines accurately account for activation of the ascending arousal system, then it can be speculated that exercise intensities below the CT do not induce activation of the ascending arousal system [6, 7]. However, it is too early to arrive at a such a conclusion: our sequential translational studies from rodents to humans have shown that even exercise below the LT immediately elicits hippocampal activation and post-exercise task-related prefrontal and hippocampal activation coinciding with increased psychological arousal [17–19]. This leads us to the hypothesis that the ascending arousal system, including the LC, is robustly activated even with very-light-/light-intensity exercise below the CT compared with a resting control. However, there is little physiological evidence that very-light-/light-intensity exercise activates the ascending arousal system.

Human evidence is lacking due to insufficient techniques that can be used to measure the ascending arousal system during dynamic exercise. Thus, pupillometry could be an important tool as a biomarker of arousal system activation during exercise. Beyond its visual function, pupil dilation, part of the autonomic nervous system, can sensitively reveal LC neuron activation [20–22]. In rodents, pupil dynamics allow the tracking of the activation of LC axon terminals in the cortex and of the cortical physiological signature synchronizing with locomotion in real time [23–25]. The pharmacological blocking of LC-arousal activation reduced pupil dilation during locomotion [26]. While pupillometry has recently become popular in neuroscience as a reliable indicator of the ascending arousal system [27, 28], it has not been applied much in exercise physiology. Several previous studies suggest that physical exercise leads to dilation of the human pupil [29–33], but most of these were based on handgrip exercise, which is a static and localized exercise [29, 32, 33]. For dynamic aerobic exercise, Hayashi and colleagues found that pupil diameter increases with exercise intensity during graded exercise up to the equivalent of moderate-intensity exercise as predicted by heart rate (HR)[31]. It should be noted, however, that their findings were limited to an exercise range of rest to moderate intensity and did not include maximal exercise. In addition, metabolic and arousal responses corresponding to graded-exercise-induced pupil dilation were not examined. It is necessary to accurately identify the pupil dilation corresponding to each exercise intensity with reference to oxygen intake (${\dot{V}}_{{\text{O}}_{2}}$) (the gold standard for measuring exercise intensity), carbon dioxide output (${\dot{V}}_{{\text{CO}}_{2}}$), and minute ventilation (${\dot{V}}_{\text{E}}$) (used for inspecting VT), and corresponding to arousal response to grow into a new physiological indicator that could reveal the brain’s state of arousal during exercise. This is essential information for identifying the pupil response that denotes activation of the ascending arousal system with very-light-/light-intensity exercise below the VT, which was our target.

To this end, the current study examined the exact exercise intensity that leads to pupil dilation to provide physiological evidence that very-light-/light-intensity exercise activates the ascending arousal system. First, we hypothesized that the pupil dilates with exercise intensity and possibly has inflection points, such as do the CT, VT, and LT. In addition, we hypothesized that the pupil dilates robustly with the arousal response with very-light-/light-intensity exercise below inflection points corresponding to the CT, VT, and LT.

## Methods

### Participants

Twenty-nine healthy young males, with no self-reported history of neurological or psychiatric disorders, were recruited for the study. The following exclusion criteria were used: taking medication that affects the central nervous and/or endocrine systems (*N* = 1), failed to achieve ${\dot{V}}_{{\text{O}}_{\text{2peak}}}$ criteria (*N* = 1), and failed to observe exercise intensity during the “very-light-intensity” condition (*N* = 1). Ultimately, 26 participants were included. Post-hoc sensitivity analysis, computed using G*Power 3.1, performed based on the current sample size (26 participants) with 80% power and multiple testing corrected alpha of 0.05 demonstrated sufficient sensitivity for detecting *t* test differences exceeding *d* = 0.81 (with a two-tailed alpha). Table 1 depicts the characteristics of the participants.

**Table 1:** Participant details

Measure	Mean ± SD
Sample size (*n*)	26
Age (years)	22.4 ± 1.5
Height (cm)	171.3 ± 5.5
Weight (kg)	71.7 ± 14.1
BMI (kg/m^2^)	24.3 ± 4.2
Graded exercise test	
${\dot{ V}}_{{\text{O}}_{\text{2peak}}}$(ml/min/kg)	43.3 ± 7.4
HR_peak_ (bpm)	179.8 ± 12.0
WR_peak_ (watt)	249.6 ± 31.1
RPE_peak_ (score)	19.8 ± 0.4

Values are presented as mean ± SD

BMI, body mass index; ${\dot{V}}_{{\text{O}}_{\text{2peak}}}$, peak oxygen uptake; HR, heart rate; WR, work rate; RPE, ratings of perceived exertion

### Procedure

After confirmation of the participants’ physical conditions, they performed a graded exercise test. Participants were asked to refrain from exercise and the consumption of alcohol for at least 24 h prior to the experiment and caffeine on their experimental day so as to control for outside factors. The intensity of illumination in the room was maintained at about 1440–1530 lx, measured with an illuminance meter placed on a central desk in the experiment room.

### Graded exercise test protocol

The seat height of the cycle ergometer (Corival Recumbent, Lode, The Netherlands) was adjusted to allow each participant to sit comfortably in a semi-supine position. After resting for 4 min and then warming up for 3 min at 30 W, the workload was increased by 20 W per min constantly and continuously until maximal effort was reached. Exhaled gas was analyzed using a gas analyzer (Aeromonitor AE280S, Minato Medical Science, Japan). Ventilation parameters, ${\dot{V}}_{{\text{O}}_{2}}$, ${\dot{V}}_{{\text{CO}}_{2}}$, and ${\dot{V}}_{\text{E}}$, were measured in expiration mode at a sampling rate of every 5 s. HR was recorded (Polar RS800CX, Polar, Finland) throughout. These sampling data were averaged every 15 s for further analysis. The pedaling rate was maintained at 60 rpm. All participants performed exercise until they could no longer maintain a pedal rotation speed of 60 rpm. ${\dot{V}}_{{\text{O}}_{2}{\text{peak}}}$ was determined when at least two of the following criteria were satisfied: (1) the respiratory exchange ratio exceeded 1.10; (2) 90% of age-predicted peak HR (220—age) was achieved; and (3) Borg Rating of Perceived Exertion (RPE) reached 18 or more [34].

### Measurement of psychological arousal and rating of perceived exertion

Participants were asked about their psychological arousal and perceived exertion every minute. Psychological arousal was measured using the two-item version of the Two-Dimensional Mood Scale (TDMS-2), which is a short version of the eight-item TDMS (TDMS-8) [3, 18, 19, 35, 36]. Participants were asked to indicate their state at the time on an 11-point scale from − 5 to + 5. Arousal scores were calculated by subtracting the Stability score (− 5 indicates "irritated" or "not at all calm," 0 indicates neutral, and + 5 indicates "relaxed" or "very calm") from the Vitality score (− 5 indicates "lethargic" or "not at all energetic," 0 indicates neutral, and + 5 indicates "lively" or "full of energy”). Previously, a high correlation between the TDMS-2 and TDMS-8 was confirmed in a mild-exercise condition (*N* = 168; *r* = 0.71)[37]. Perceived exertion was measured using the Borg Rating of Perceived Exertion (RPE). The TDMS and RPE questionnaires were presented on a screen attached to the ergometer 70 cm from the participant. The TDMS and RPE screens alternated every 30 s.

### Acquisition and preprocessing of pupillometry data

Participants’ pupil diameters were recorded using a screen-based eye-tracker (Tobii pro nano, Tobii AB, Danderyd, Sweden). The eye-tracking data were recorded continuously from resting before exercise to the end of the exercise. This measurement method allows for the monitoring of longer-lasting pupil dilation during exercise compared to the baseline pupil diameter at rest. Participants were instructed to keep their eyes on the screen (background: RGB: 120, 120, 120, letters: RGB: 0, 0, 0) in front of them during the session, not just when answering TDMS and RPE questions. During the graded exercise, participants were asked not to move their heads for as long as they were able. The data were sampled at 60 Hz. The analysis software (Tobii Pro Lab, Tobii AB, Danderyd, Sweden) automatically removed missing and invalid pupil data in cases, where the participant blinked or looked away from the screen. Raw data were taken at an average of every 15 s as were the gas sampling data. Pupil detection rates tended to decrease around near-maximal to maximal intensity exercise (53% compared to rest), because participants blinked, moved their faces or looked away from the screen. However, there was rarely a complete loss of pupil detection for 15 consecutive seconds (99.6% acquisition rate) and the sample number for raw pupil data used to calculate the 15-s mean was 281 ± 148 at this intensity. As explained in the data analysis section below, the 15-s mean data were averaged once more by intensity category. In this way, a sufficient sample size was maintained to produce a valid average of pupil diameter.

### Data analysis

First, we examined how pupils dilated in response to increased exercise intensity. Exercise intensity was categorized according to American College of Sports Medicine (ACSM) criteria based on %${\dot{V}}_{{\text{O}}_{\text{2peak}}}$, the gold standard for determining exercise intensity. The data are divided into the following intensity intervals: rest, very-light (< 37% ${\dot{V}}_{{\text{O}}_{\text{2peak}}}$ including warm-up), light (37–45% ${\dot{V}}_{{\text{O}}_{\text{2peak}}}$), moderate (46–63% ${\dot{V}}_{{\text{O}}_{\text{2peak}}}$), vigorous (64–90% ${\dot{V}}_{{\text{O}}_{\text{2peak}}}$), and near maximal to maximal (> 91% ${\dot{V}}_{{\text{O}}_{\text{2peak}}}$) for each individual [38]. Based on these criteria, we examined the differences between intensities using repeated measures one-way ANOVA and post-hoc multiple comparisons with Holm–Sidak correction.

In addition, the Δ pupil diameter between each intensity was calculated [i.e., (very-light–rest), (light–very-light), (moderate–light), (vigorous–moderate), (near max/max–vigorous)] to examine the exercise intensity intervals at which pupil dilation was prominent. The pupil dilation values were adjusted by dividing them by the corresponding %${\dot{V}}_{{\text{O}}_{\text{2peak}}}$ changes and determining the increase in dilation for each 1% ${\dot{V}}_{{\text{O}}_{\text{2peak}}}$ increase between intensity intervals. For comparison of the Δ pupil diameter changes between intensities, repeated measures one-way ANOVA and post-hoc multiple comparisons with Holm–Sidak correction were conducted. For reference, %${\dot{V}}_{{\text{O}}_{\text{2peak}}}$-adjusted Δ HR and Δ ${\dot{V}}_{\text{E}}$ were calculated in the same way; HR is a linearly increasing index, while ${\dot{V}}_{\text{E}}$ has an inflection point (VT).

Next, we examined the association between interindividual differences in pupil diameter and psychological arousal level, which increased with very-light-intensity exercise. The change from rest was calculated [i.e., (very-light–rest)], and a Pearson correlation analysis of the increase in arousal and pupil diameter was performed. As an additional exploratory analysis, the changes between one rank of intensity and the previous rank were calculated [i.e., (light–very-light), (moderate–light), (vigorous–moderate), (near-max/max–vigorous)], and correlation between pupil diameter and psychological arousal level at other exercise intensities were tested. Statistical significance was set a priori at *P* < 0.05. Statistical analyses were performed using Prism 9 (Version 9.1.1).

## Results

### Respiratory gas parameter, heart rate, and pupil changes

**Fig. 1:**
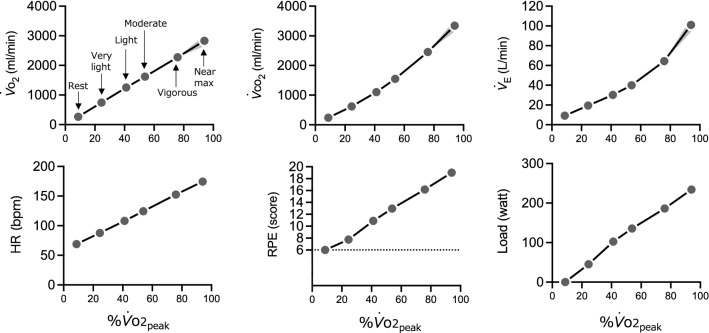
${\dot{V}}_{{\text{O}}_{2}}$, ${\dot{V}}_{{\text{CO}}_{2}}$, ${\dot{V}}_{\text{E}}$, HR, RPE, and Load. The data for each intensity are plotted against %${\dot{V}}_{{\text{O}}_{\text{2peak}}}$. on the *x*-axis and each parameter on the *y*-axis. The plots from left to right show rest, very-light, light, moderate, vigorous, and near maximal/maximal intervals. ${\dot{V}}_{{\text{O}}_{2}}$, oxygen intake; ${\dot{V}}_{{\text{CO}}_{2}}$, carbon dioxide output; ${\dot{V}}_{\text{E}}$, minute ventilation; HR, heart rate; RPE, ratings of perceived exertion. Data are presented as mean. The gray bands represent 95% CI, although some are too small to clearly visualize. The horizontal error bars (95% CI) cannot be visualized as all were smaller than 1.0% ${\dot{V}}_{{\text{O}}_{\text{2peak}}}$

**Fig. 2:**
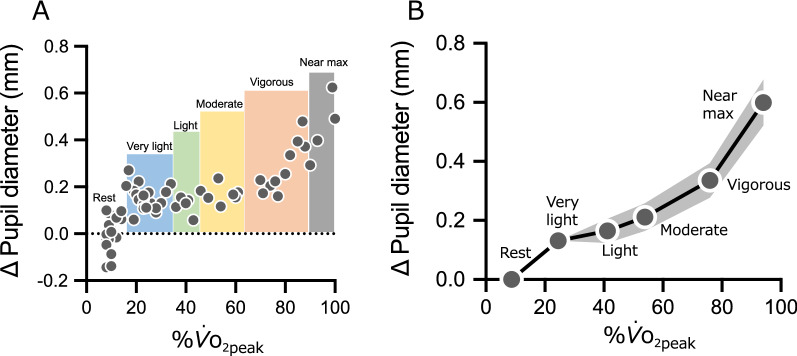
**A** Typical example of the change in pupil diameter. **B** Change in pupil diameter compared to rest. The data for each intensity are plotted for %${\dot{V}}_{{\text{O}}_{\text{2peak}}}$. (*x*-axis) and Δ pupil diameter (*y*-axis). The plots from left to right show rest, very-light, light, moderate, vigorous, and near maximal/maximal. Uncorrected pupil diameter data were used for statistical analysis. Data are presented as mean. The gray bands represent 95% CI, although some are too small to clearly visualize. The horizontal error bars (95% CI) cannot be visualized as all were smaller than 1.0%${\dot{V}}_{{\text{O}}_{\text{2peak}}}$

${\dot{V}}_{{\text{O}}_{2}}$, ${\dot{V}}_{\text{E}}$, ${\dot{V}}_{{\text{CO}}_{2}}$, HR, and RPE increased with graded exercise in an intensity-dependent manner (Fig. 1). The repeated measures one-way ANOVA for pupil diameter revealed significant differences between intensities (*F* (2.17, 54.32) = 119.2, *P* < 0.001). The post-hoc test showed significant differences between all intensities (all *t* (25) > 2.91, *P* < 0.008, Holm–Sidak corrected) (Fig. 2).

**Fig. 3:**
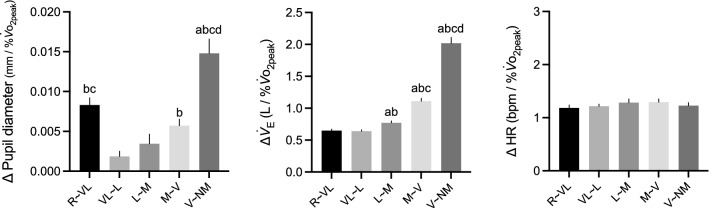
Δ%${\dot{V}}_{{\text{O}}_{\text{2peak}}}$-adjusted Δ pupil diameter, Δ ${\dot{V}}_{\text{E}},$ and Δ HR for each intensity interval. R, rest; VL, very-light; L, light; M, moderate; V, vigorous; NM, near maximal/maximal; ${\dot{V}}_{{\text{O}}_{\text{2peak}}}$, peak oxygen uptake; HR, heart rate; ${\dot{V}}_{E}$, minute ventilation; ^a^*P* < 0.05 greater than R-VL; ^b^*P* < 0.05 greater than VL-L; ^c^*P* < 0.05 greater than L-M; ^d^*P* < 0.05 greater than M-V. Data are presented as mean ± SE

Next, the prominent exercise intensity at which pupil dilation increased was examined (Fig. 3). The repeated measures one-way ANOVA for %${\dot{V}}_{{\text{O}}_{\text{2peak}}}$-adjusted Δ pupil diameter revealed significant differences between intensities (*F* (2.47, 61.77) = 18.64, *P* < 0.001). The post-hoc test showed that the Δ pupil diameter between rest and very-light significantly increase compared with that between very-light and light, and light and moderate. In addition, Δ pupil diameter between moderate and vigorous significantly increased compared to that at very-light to light, and Δ pupil diameter between vigorous and near-max/max significantly increased compared to that between rest and very-light, very-light and light, light and moderate, and moderate and vigorous. %${\dot{V}}_{{\text{O}}_{\text{2peak}}}$-adjusted Δ HR exhibited no significant differences between intensities (*F* (3.30, 82.59) = 0.70, *P* = 0.57), which indicates that Δ HR increased linearly with increasing ${\dot{V}}_{{\text{O}}_{2}}$. In contrast, %${\dot{V}}_{{\text{O}}_{\text{2peak}}}$-adjusted Δ ${\dot{V}}_{\text{E}}$ increased exponentially (*F* (1.51, 37.76) = 199.9, *P* < 0.001).

### Association between inter-individual differences in pupil change and arousal levels induced by very-light-intensity exercise

**Fig. 4:**
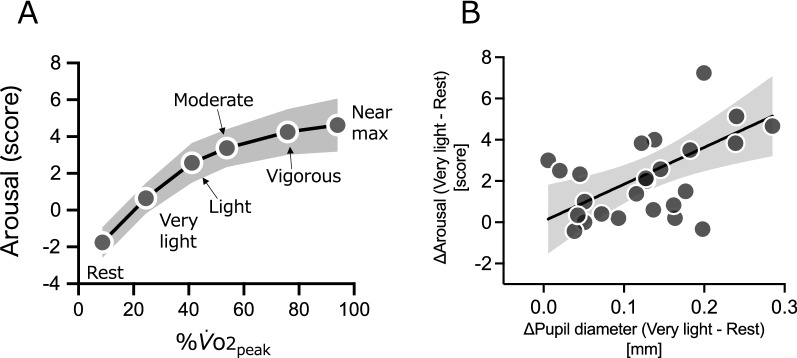
**A** Arousal levels. The data at each intensity (*y*-axis) are plotted against %${\dot{V}}_{{\text{O}}_{\text{2peak}}}$. (*x*-axis). The plots, from left to right, show rest, very-light, light, moderate, vigorous, and near maximal/maximal intervals. Data are presented as mean. The gray band represents 95% CI. The horizontal error bars (95% CI) cannot be visualized as all were smaller than 1.0%${\dot{V}}_{{\text{O}}_{\text{2peak}}}$. **B** Association between Δ pupil diameter (very-light–rest) and Δ arousal (very-light–rest). The black line represents linear regression and gray band represents 95% CI

The repeated measures one-way ANOVA for psychological arousal revealed significant differences between intensities (*F* (1.72, 43.0) = 42.68, *P* < 0.001). The post hoc test showed significant differences between all intensities (all *t* (25) > 3.33, *P* < 0.006, Holm–Sidak corrected), with the exception of those between vigorous and near-max/max (*t* (25) = 1.56, *P* = 0.13, Holm–Sidak corrected) (Fig. 4A). There was a significant positive correlation between Δ pupil diameter [very-light–rest] and the Δ arousal level [very-light–rest] (*r* = 0.55, *P* = 0.0034)(Fig. 4B). On the other hand, there were no significant correlations between Δ pupil diameter and Δ arousal level in other adjacent intensities (Δ [light–very-light]: *r* = − 0.18; Δ [moderate–light]: *r* = 0.11; Δ [vigorous–moderate]: *r* = 0.08 and Δ [near-max/max–vigorous]: *r* = − 0.08; all *P* > 0.05).

## Discussion

This study aimed to delineate the pupil dilation that occurs during graded exercise ranging from very-light intensity, to obtain a comprehensive understanding of arousal system activation in relation to exercise intensity. The results reveal that pupils dilated with very-light-/light-intensity exercise, and this was then correlated with an increase in psychological arousal states. At stronger intensities, pupils dilated drastically when participants were pedaling all out, as with ${\dot{V}}_{\text{E}}$. The unique pupil dilation pattern observed during the initial stage of graded exercise may explain the beneficial workings of the ascending arousal system even with very-light-/light-intensity exercise.

We observed that graded exercise leads to pupil dilation in an exercise-intensity-dependent manner. This result replicates and expands upon the previous study by Hayashi and colleagues [31]. They similarly found significant intensity-dependent pupil dilation at intensities from very-light to moderate referenced, in their study, against HR increase. The current study replicated their results based on %${\dot{V}}_{{\text{O}}_{{\text{2pe}}{\text{ak}}}}$, the gold standard for measuring exercise intensity. In addition, the current study examined the intensities at which the pupil is prominently dilated and found that the increase in dilation is exponential in an exercise-intensity-dependent manner as is ${\dot{V}}_{\text{E}}$. These results suggest that, such as VT, pupil dilation has inflection points, which we name the pupil dilation threshold (PDT), rather than a linear trajectory [note that the intensity at which the PDT occurs may differ between individuals (Additional file 1: Fig. S1)]. Although we did not measure circulating blood parameters to minimize external stimuli that may affect mood and pupil diameter via arousal in the present study, the pupil dilation profile we have observed seems to correspond to increasing circulating catecholamines as found in past studies [9, 39]. Interestingly, there were major differences between changes in pupil dilation and changes in ${\dot{V}}_{\text{E}}$ for the rest/very-light-intensity exercise comparison. The change in Δ%${\dot{V}}_{{\text{O}}_{\text{2peak}}}$-adjusted pupil dilation for [rest to very-light-intensity] was greater than that for [very-light to light], [light to moderate] intensities. This demonstrates not only that pupil diameter has an inflection-like VT, but also that the pupil clearly dilates with very-light-intensity exercise below the PDT.

We then considered psychological arousal, which was monitored during graded exercise, to test whether pupil dilation functioned as an indicator of activation of the ascending arousal system. Psychological arousal increased in an intensity-dependent manner with graded exercise beginning with very-light-intensity exercise, which is consistent with previous studies, including our own [1, 18, 19, 40]. Our previous neuroimaging studies have shown the functional significance of very-light-intensity exercise inducing a psychological arousal response; this is associated with subsequent enhanced executive function with lateral prefrontal cortex activation and memory function with hippocampal activation [18, 19]. The present results demonstrate that the pupil dilation induced by very-light-intensity exercise correlates significantly with inter-individual differences in psychological arousal with very-light-intensity exercise. Therefore, it is possible that very-light-intensity exercise activates the ascending arousal system in synchronicity with pupil dilation and that this is expressed through psychological arousal. This psychological examination supports our hypothesis that the initial stage of pupil dilation functions as a physiological marker for activation of the ascending arousal system during very-light to light exercise.

Although the pupil may be an important marker for explaining body–brain interactions during exercise, further studies are needed to understand the neural circuit. Pupil dilation during exercise is possibly mediated by signals from the breathing control center neurons, the cortex and the afferent nerves in working muscles (e.g., metaboreflex) to the brainstem, including LC neurons [21, 32, 41]. In particular, the physiological mechanism for pupil dilation during stress-free very-light-intensity exercise below LT/VT/CT is an interesting question for future study.

This study has some limitations with regard to generalization. First, it included only healthy, young male participants to avoid potential effects of the female cycle phase and to observe the same graded load protocols in participants with homogeneous fitness levels. Second, the results may have been influenced by the brightness of the room. As illumination affects pupil diameter, if the study was conducted in a dimly lit room (e.g., 10 lx illumination), pupil diameter may encounter a ceiling effect rendering the effect of exercise undetectable [30]. Finally, the graded exercise method has methodological limitations. Accumulation effects and time gaps may distort the results. Thereby, time-controlled validation would strengthen the current findings.

## Conclusions

**Fig. 5:**
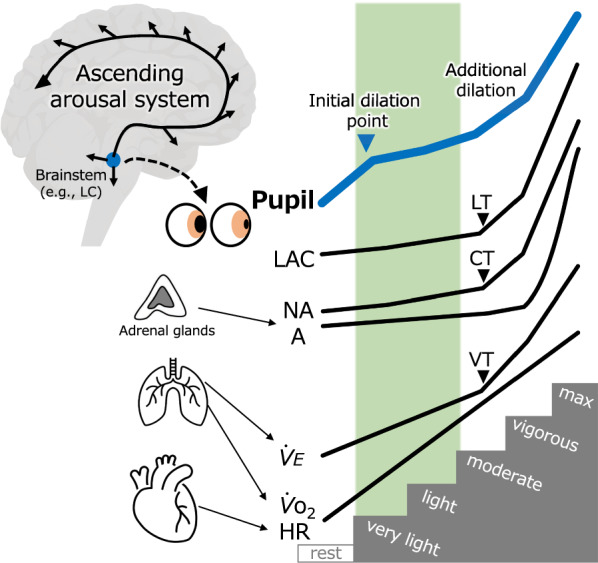
Schematic illustrating intensity-dependent pupil and physiological/biochemical responses. In contrast to other physiological/biochemical parameters, which exhibit pronounced increases at the LT/VT/CT points, the pupils dilate markedly not only around the LT/VT/CT points or at higher exercise intensities (additional dilation, i.e., PDT), but at very-light-intensity exercise (initial dilation point). This profile may indicate early activation of the ascending arousal system and explain the effects of exercise below the LT/VT/CT (area of the vertical green bar) on arousal states. Parameters that the current study did not measure (LAC, NA and A) were drawn based on previous studies and reviews [9, 10, 42, 43]. LAC, blood lactate; NA, blood noradrenaline; A, blood adrenaline; ${\dot{V}}_{\text{E}}$, minute ventilation; ${\dot{V}}_{{\text{O}}_{2}}$, oxygen intake; HR, heart rate; PDT, pupil dilation threshold; LT, lactate threshold; VT, ventilation threshold; CT, catecholamine threshold; LC, locus coeruleus

Exercise-intensity-dependent pupil dilation was found to have a threshold-like profile (Fig. 5). Specifically, not only does pupil diameter have an inflection, like that of VT, but the pupil also dilates at the initial exercise stage (i.e., very-light-intensity), which corresponds to enhanced psychological arousal. This unique pupil dilation pattern provides physiological evidence of a beneficial arousal mechanism with very-light-intensity exercise that could not be explained by previously examined peripheral markers. Pupillometry during exercise may become applicable to exercise prescription targeting adequate arousal states in the brain.

### Supplementary Information

**Additional file 1: Figure S1.** Individual data for Δ pupil diameter.

## Data Availability

The data sets generated during and/or analyzed during the current study are available from the corresponding author on reasonable request.

## Abbreviations

CT: Catecholamine threshold
LT: Lactate threshold
VT: Ventilatory threshold
LC: Locus coeruleus
HR: Heart rate
$${\dot{V}}_{{\text{O}}_{2}}$$: Oxygen intake
${\dot{V}}_{{\text{CO}}_{2}}$: Carbon dioxide output
$${\dot{V}}_{\text{E}}$$: Minute ventilation
WR: Work rate
RPE: Ratings of perceived exertion.
ACSM: American College of Sports Medicine
TDMS-2: Two-item version of the Two-Dimensional Mood Scale
TDMS-8: Eight-item version of the Two-Dimensional Mood Scale
R: Rest
VL: Very-light
L: Light
M: Moderate
V: Vigorous
NM: Near maximal/maximal
PDT: Pupil dilation threshold
LAC: Blood lactate
NA: Blood noradrenaline
A: Blood adrenaline
