# Supplementary material for: Pupil-linked arousal with very light exercise: pattern of pupil dilation during graded exercise
Source: J Physiol Sci. 2022 Sep 24;72:23. doi: 10.1186/s12576-022-00849-x (PMC10717467; doi:10.1186/s12576-022-00849-x)
Supplement: Supplementary file 1 — Additional file 1: Figure S1. Individual data for Δ pupil diameter. [file 12576_2022_849_MOESM1_ESM.pdf]

Additional file 1 for

Pupil-linked arousal with very light exercise: Pattern of pupil dilation during graded exercise

Ryuta Kuwamizu, Yudai Yamazaki, Naoki Aoike, Genta Ochi, Kazuya Suwabe & Hideaki Soya

Individual data for pupil dilation

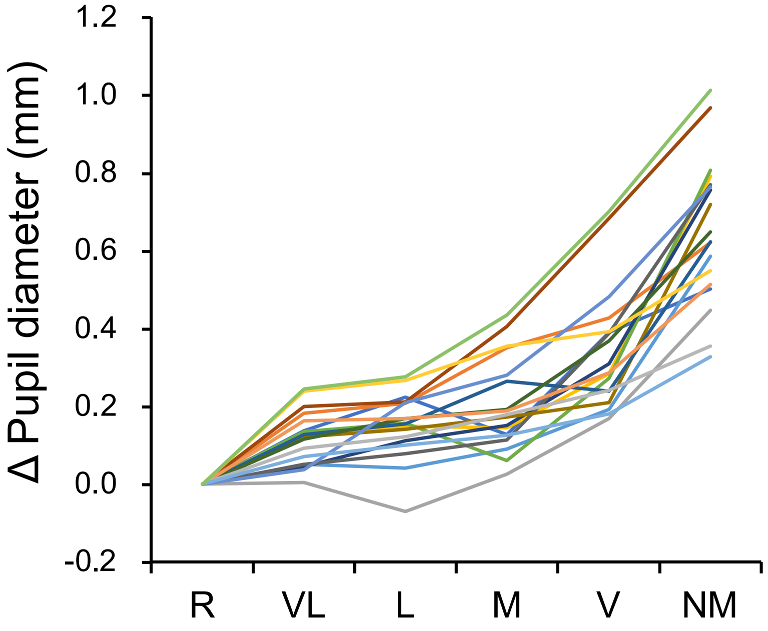

Fig. S1. Individual data for Δ pupil diameter. Although it appears as though there are individual differences, there is a threshold-like response (i.e., pupil dilation threshold: PDT) when considered as an average. Further research is needed to better understand these individual differences.

R: rest; VL: very light; L: light; M: moderate; V: vigorous; NM: near maximal/maximal
